# Liquid biopsy in hematological malignancies: current and future applications

**DOI:** 10.3389/fonc.2023.1164517

**Published:** 2023-04-20

**Authors:** Donatella Talotta, Mohammad Almasri, Chiara Cosentino, Gianluca Gaidano, Riccardo Moia

**Affiliations:** Division of Hematology, Department of Translational Medicine, Università del Piemonte Orientale, Novara, Italy

**Keywords:** liquid biopsy, DLBCL, Hodgkin lymphoma, myeloid neoplasia, precision medicine

## Abstract

The assessment of the cancer mutational profile is crucial for patient management, stratification, and therapeutic decisions. At present, in hematological malignancies with a solid mass, such as lymphomas, tumor genomic profiling is generally performed on the tissue biopsy, but the tumor may harbor genetic lesions that are unique to other anatomical compartments. The analysis of circulating tumor DNA (ctDNA) on the liquid biopsy is an emerging approach that allows genotyping and monitoring of the disease during therapy and follow-up. This review presents the different methods for ctDNA analysis and describes the application of liquid biopsy in different hematological malignancies. In diffuse large B-cell lymphoma (DLBCL) and Hodgkin lymphoma (HL), ctDNA analysis on the liquid biopsy recapitulates the mutational profile of the tissue biopsy and can identify mutations otherwise absent on the tissue biopsy. In addition, changes in the ctDNA amount after one or two courses of chemotherapy significantly predict patient outcomes. ctDNA analysis has also been tested in myeloid neoplasms with promising results. In addition to mutational analysis, liquid biopsy also carries potential future applications of ctDNA, including the analysis of ctDNA fragmentation and epigenetic patterns. On these grounds, several clinical trials aiming at incorporating ctDNA analysis for treatment tailoring are currently ongoing in hematological malignancies.

## Introduction

The assessment of the cancer mutational profile is nowadays crucial for patient management, stratification, and therapeutic decisions. At present, in hematological malignancies with solid masses, such as lymphomas, tumor genomic profiling is generally performed on the tissue biopsy. Different genomic and molecular analyses on tissue biopsy complement histological diagnosis for the detection of molecular biomarkers with prognostic and therapeutic implications (1–3). However, the current approach for accessing tumor material consists of invasive procedures with limitations with respect to feasibility and the collection of serial samples for real-time monitoring. Moreover, tissue biopsy is usually confined to one single tumor site, thus limiting a comprehensive characterization of the tumor genome, which might vary among different anatomical sites (4).

**Figure 1:**
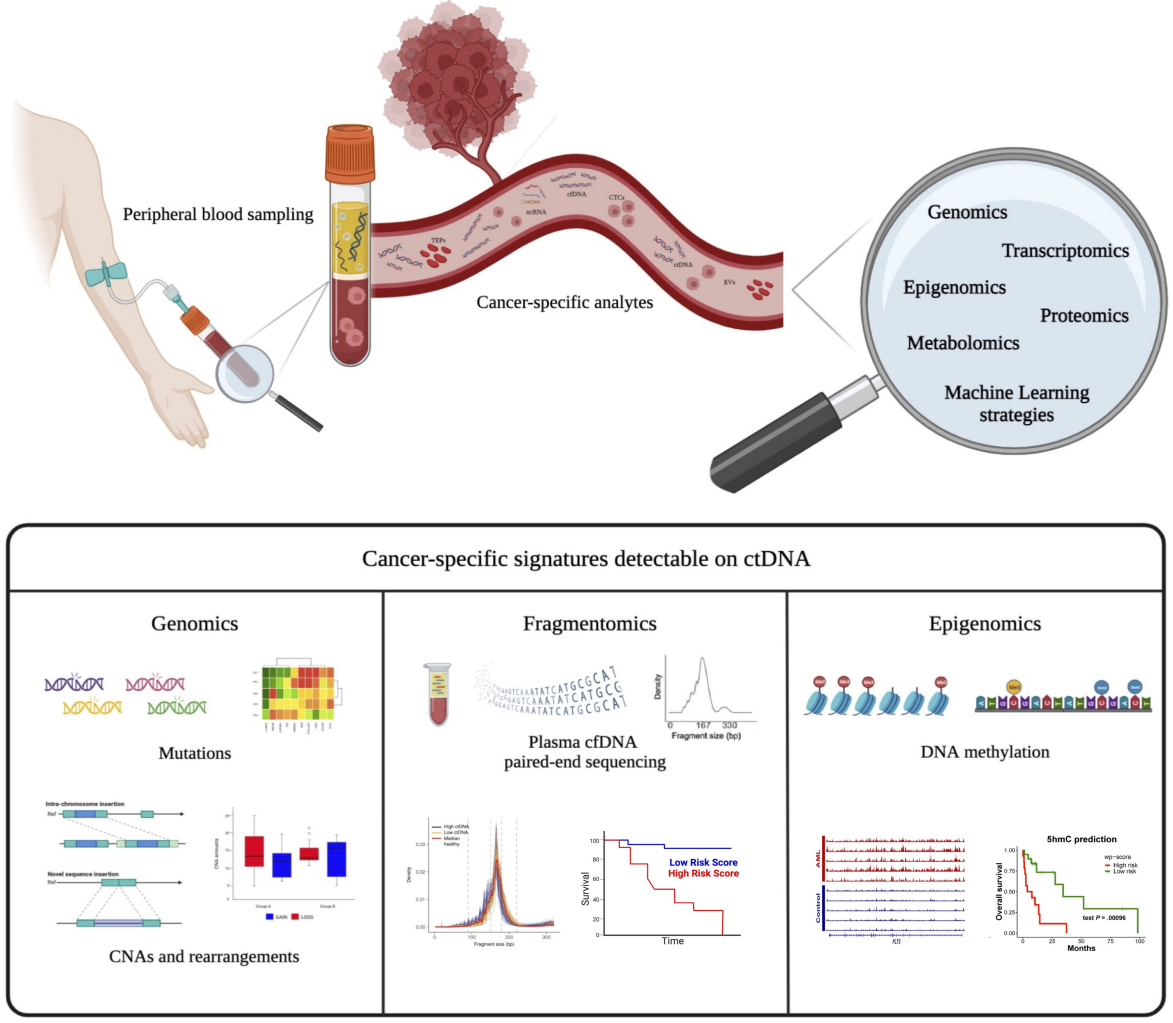
Liquid biopsy as a tool for identifying various cancer-specific analytes. Tumor-specific circulating analytes potentially detected by liquid biopsy include cell-free DNA (cfDNA) and/or circulating tumor DNA (ctDNA), circulating cells (CTCs), extracellular vesicles (EVs), and tumor-educated platelets (TEPs). Different cancer-specific genomic signatures can be revealed from ctDNA, including mutations, copy number alterations (CNAs), fragmentomics, and DNA methylation patterns. These biological data could be integrated using innovative bioinformatic pipelines to improve precision medicine.

**Figure 2:**
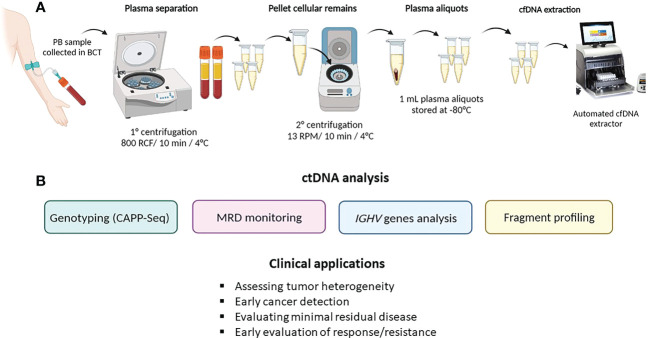
ctDNA collection method and analysis. **(A)** describes ctDNA isolation and extraction from peripheral blood (PB); **(B)** summarizes the main ctDNA applications in hematological malignancies.

Liquid biopsy is an emerging approach for characterizing tumors through the isolation and analysis of different cancer-derived components released in any body fluids (i.e., blood, urine, liquor, or others) (**Figure 1**). These “clues” released from tumors may be represented by circulating tumor cells (CTCs), circulating cell-free nucleic acids, exosomes, or tumor-educated platelets (TEPs) (5). In the context of hematological malignancies, liquid biopsy is a minimally invasive and real-time procedure that can potentially overcome the intrinsic limitations of tissue biopsies, which expose patients to procedural risks and cannot account for spatial intratumor heterogeneity. Different synchronous sources of tumor DNA complement each other in informing on driver gene mutations harboring potential prognostic and/or predictive value (6). The analysis of circulating tumor DNA (ctDNA) from plasma represents the most used application of liquid biopsy in hematological malignances for disease genotyping and for dynamic monitoring of the disease response during therapy (7, 8). The role of liquid biopsy for minimal residual disease (MRD) monitoring has been extensively reviewed elsewhere (9). In the present review, we will discuss the current application of liquid biopsy in lymphoid and myeloid neoplasms and the potential future applications that may allow a more personalized treatment approach in every single patient (**Figure 2**).

## Methods for ctDNA detection and analysis

### Circulating cell-free DNA and circulating tumor DNA

The definition of circulating cell-free DNA (cfDNA) refers to short DNA fragments (180-200 base pairs) shed by apoptotic or necrotic cells in the bloodstream (10). In healthy subjects, the concentration of cfDNA in plasma ranges from 1 to 16.8 ng/ml, has a short half-life (approximately 2 h), and can increase following conditions such as physical exercise, acute trauma, stroke, and infection (11). In cancer patients, cfDNA levels are significantly higher than in healthy individuals, since apoptotic and necrotic remains within the tumoral mass are not cleared efficiently by phagocytes, leading to the accumulation of cellular debris, including cfDNA, that is subsequently released into the circulation (7). In these patients, cfDNA comprises molecules that are derived both from healthy cells and tumor cells. To differentiate the origin, the fraction of cfDNA derived from tumors cells by multiple shedding mechanisms (apoptosis, necrosis, and secretion) is known as circulating tumor DNA (ctDNA) (7, 12). In lymphoma patients, ctDNA levels vary greatly across different lymphoma subtypes and reflect tumor type, clinical stage, and tumor burden. Overall, the mean concentration of ctDNA in lymphoma patients is 30 ng/ml (13).

### ctDNA collection

As previously stated, ctDNA can be isolated and analyzed from any fluid of the body. Given the high accessibility of peripheral blood, the watery fluid portion, namely, the serum or plasma, represents the most frequent source of ctDNA. Despite the fact that the amount of ctDNA fragments is similar in both serum and plasma (14), the latter represents the optimal specimen type for ctDNA analysis because the amount of cfDNA released by normal leukocytes that dilutes the ctDNA is lower in plasma than in serum. Blood samples should be collected in tubes containing K_2_EDTA and processed for plasma separation within 6 h in order to avoid contamination derived from genomic DNA released by leukocytes (15, 16). Alternatively, specific blood collection tubes (i.e., Streck BCT) allow the preservation and stabilization of ctDNA for up to 14 days, thus allowing the safe shipment of samples (16, 17).

To preserve cfDNA and minimize leukocyte lysis, it is recommended to perform peripheral blood processing through a sequential pair of centrifugations prior to storage and/or cfDNA extraction (15). Studies indicate that purified plasma can be frozen and safely preserved in single-use aliquots (18, 19).

### Methods for ctDNA analysis

In hematological malignancies, ctDNA can be analyzed for the evaluation of gene mutations or the analysis of the unique immunoglobulin heavy chain (IGHV) rearrangement in cases of B-cell neoplasms (20–23).

#### Evaluation of gene mutations

Disease genotyping on ctDNA allows the identification of somatic cancer gene mutations without the need of previous analyses of the tumor biopsy, therefore working in a tissue biopsy-free manner. As previously stated, this approach has the ability to potentially identify mutations derived from different tumor sites thus allowing the characterization of the entire tumor heterogeneity (7). Ultra-deep next-generation sequencing (NGS) methods allow massive parallel sequencing of DNA molecules in a single flow cell (24) and can detect a large spectrum of genetic alterations, including point mutations, insertions/deletions, rearrangements, and copy number changes (25). The CAncer Personalized Profiling by deep sequencing (CAPP-seq) is a highly sensitive and specific targeted capture NGS method to detect tumor-specific mutations in ctDNA in molecularly heterogeneous tumors (25). CAPP-seq utilizes a disease-specific “selector,” which is a set of exonic and intronic targets that cover regions of known recurrent mutations in a particular cancer type. The subsequent amplification and sequencing of the target regions allow the quantification of ctDNA based on the detection of cancer-specific mutations (13).

#### Analysis of the IGHV rearrangement

Almost all B-cell tumors harbor a unique IGHV rearrangement that can be identified on ctDNA using polymerase chain reaction (PCR) or NGS-based techniques (20, 26, 27). In cases of unproductive IGHV rearrangement, the light chain *IGK* or *IGL* sequence could be used as a surrogate marker. However, to ensure the tumor origin of the IGHV rearrangement identified on the liquid biopsy, the IGHV rearrangement should be initially identified on the tissue biopsy. NGS-based methods, such as clonoSEQ (28), rely on universal primer sets targeting the immunoglobulin heavy or light chains. This assay is FDA-approved for MRD detection in patients with chronic lymphocytic leukemia (CLL), multiple myeloma (MM), and B-cell acute lymphoblastic leukemia (B-ALL) (29) and has been extensively used for MRD monitoring of ctDNA in lymphoma patients (20–22, 29, 30). However, these methods for immunoglobulin analysis capture only one single molecular marker and fail to detect clonal V(D)J rearrangements in ~20% of lymphoma due to the high rates of somatic hypermutation (SHM) (20, 22, 24).

## Applications in hematological malignancies

### Diffuse large B-cell lymphoma

Diffuse large B-cell lymphoma (DLBCL) is a highly heterogeneous disease both from a molecular and clinical standpoint. Molecular heterogeneity is present also within the same patient, and different anatomical sites may harbor different genetic lesions. On these grounds, liquid biopsy has been demonstrated to be able to detect mutations otherwise absent in the single tissue biopsy. Different studies have demonstrated that lymphoma-related genetic alterations can be detected from ctDNA using CAPP-seq even without a baseline tumor tissue biopsy (4, 31, 32). The sensitivity and specificity of targeted gene mutation analysis in ctDNA *versus* tissue biopsy in DLBCL patients have been assessed in several independent CAPP-seq studies (4, 31). The percentage of mutations (i.e., true-positive rate) identified both in the tissue biopsy and in ctDNA ranges between 95% and 99%. Moreover, liquid biopsy is able to detect a fraction of mutations (approximately 15%-20%) that are not otherwise identified in the lymph node biopsy. Conversely, only a small fraction of mutations (1% to 5%) characterized by low allelic abundance was identified in the lymph node biopsy only and was not detected in ctDNA (i.e., false-negative rate) (4, 31).

Although the majority of DLBCL patients are cured with the first-line chemoimmunotherapy R-CHOP (rituximab, cyclophosphamide, doxorubicin, vincristine and prednisone), novel emerging therapies have been recently approved as additional treatment options (33, 34). Despite these advancements, a considerable fraction of DLBCL patients (~40%) still relapse or are refractory (35, 36). The identification of clinical and molecular biomarkers that better identify high-risk patients may improve the outcome and the cure rates of DLBCL patients. At present, the therapy response criteria in lymphomas do not include MRD markers at the end of the therapy (37). The quantification of ctDNA during and after therapy represents a potential opportunity to evaluate MRD in DLBCL.

Different techniques have been used to evaluate MRD in DLBCL, using both IGHV rearrangement and gene mutations using CAPP-Seq (21, 30, 38, 39). By using a quantitative high-throughput method to analyze IGHV rearrangements, ctDNA measurement has been shown to detect disease progression months before conventional imaging in DLBCL (21). The other approach based on gene mutations relies on the CAPP-seq as a tool for the quantification of ctDNA at baseline and during the course of R-CHOP therapy. The 2-log drop from baseline ctDNA after one cycle [early molecular response (EMR)] and the 2.5-log decrease after two cycles [major molecular response (MMR)] were identified as the best cutoff to split patient outcome (21, 30, 38, 39). In addition, the molecular response by ctDNA remained an independent prognostic parameter for both event-free survival (EFS) and overall survival (OS) in multivariate analysis including the international prognostic index (IPI) score, cell of origin, and interim positron emission tomography combined with computed tomography (PET/CT) scan (40).

Recent advances in molecular biology and *in silico* strategies that suppress technical errors can substantially improve the sensitivity of targeted NGS-based approaches. A series of error-suppression methods have been applied to lower the background rate, resulting in a sensitivity of ~2.5:100,000 (41, 42). For example, CAPP-seq can be combined with a unique barcoding strategy with a downstream bioinformatic algorithm that largely eliminates sequencing errors and stereotypic background, facilitating ctDNA detection down to an allele frequency (AF) of ~0.002% (31, 39).

To further maximize the analytical sensitivity and to reduce background error rates, a recent innovative approach named phased variant enrichment and detection sequencing (PhasED-seq) can track two or more variants (“phased variants”) on the same strand of one single DNA molecule (43). This method lowers both the technical and the biological background signals and maintains high genome recovery, thus facilitating ctDNA monitoring down to an analytical detection limit of ~0.00005% (39, 42, 44). PhasED-seq seems particularly useful in B-cell malignancies since multiple-phased variants occur in stereotyped portions of the tumor genome mostly due to on-target and aberrant SHM driven by activation-induced deaminase (AID) (45). PhasED-seq can significantly improve sensitivity for ctDNA detection in lymphoma patients, but additional studies of PhasED-seq will be required to confirm its superiority to CAPP-seq for the identification of early relapsing patients and to identify the best clinical settings in which this technology should be used (42).

### CNS lymphomas

DLBCLs that involve the central nervous system (CNS), namely, CNS lymphomas (CNSLs), are distinguished into primary and secondary DLBCLs of the CNS. Primary CNS lymphoma (PCNSL) refers to a rare subtype of DLBCL involving the brain, eyes, leptomeninges, or spinal cord without extracranial involvement. Secondary DLBCL CNS lymphoma (SCNSL) denotes an isolated relapse of DLBCL within the CNS or synchronous CNS with systemic involvement (46–48). The diagnosis of CNSL requires invasive neurosurgical procedures that often cannot be safely performed or are delayed because of concurrent therapies (49–51). Consequently, the clinical outcomes of patients with CNSL are extremely heterogeneous, and many patients suffer recurrence or experience early mortality after first-line therapy (50). Novel diagnostic strategies and biomarkers are necessary to non-invasively detect CNSL, to better stratify patients into risk groups, and to predict therapy responses. Initially, liquid biopsy in PCNSL has been mainly explored in cerebrospinal fluid (CSF) since the low abundance of ctDNA in plasma hampered the detection of the disease (52–55). Recently, thanks to the improved sensitivity of liquid biopsy combining CAPP-seq and PhasED-seq approaches, ctDNA has been detected with a high concordant rate in both plasma and CSF in patients with CNSL (56, 57). Plasma ctDNA analysis allowed the identification of recurrent mutations within genes coding for components of the B-cell receptor signaling pathway, such as *MYD88*, *PIM1*, and *CD79b* (58, 59).

After demonstrating that ctDNA analysis can genotype CNSL, the potential prognostic value of ctDNA before and after therapy was evaluated. Patients with positive ctDNA in pretreatment plasma also had significantly shorter PFS and OS when adjusted in a multivariate manner for established clinical and radiographic risk factors. Moreover, similar to DLBCL, measurable residual disease detection by plasma ctDNA during treatment identified patients with a particularly poor prognosis following curative-intent immunochemotherapy (56).

### Hodgkin lymphoma

The low abundance of malignant Hodgkin/Reed-Sternberg (HRS) cells in biopsy samples (0.1%-3%) represents the main obstacle to the comprehensive genomic characterization of Hodgkin lymphoma (HL) (60). To overcome the limitations of tissue-based genotyping, plasma ctDNA was identified as a reliable source of tumor DNA for HL mutational profiling (32, 61, 62). The levels of cfDNA in HL patients are two-fold higher than in healthy subjects (~3,400 *vs*. ~1,700 hGE/ml of plasma), with a median ctDNA level in HL of ~200 hGE/ml (63). Even though the tumor cell volume in cHL is 10-fold smaller than that of other aggressive lymphomas, the correlation between ctDNA levels and radiologic tumor volume in HL is remarkably similar to DLBCL (42, 61). Thus, cHL seems to release a greater amount of ctDNA than DLBCL, a fact that may be due to the high apoptotic HRS cell rate (64).

On these grounds, efforts were initially focused on HL genotyping on the liquid biopsy. Using a CAPP-seq panel made up of frequently mutated genes in HL, ctDNA successfully detected 87% of mutations identified in the microdissected HRS cells. This study also allowed us to refine the current knowledge of cHL genetics. In addition, the authors also analyzed ctDNA of patients at the time of relapse. Longitudinal ctDNA profiling allowed us to identify treatment-dependent patterns of clonal evolution in patients relapsing after chemotherapy and in patients maintaining partial remission under immunotherapy (32). Immune evasion represents one of the pathogenetic hallmarks of HL and efforts have also been made to identify genetic lesions of immune checkpoint genes on ctDNA. Genomic profiling of cfDNA can precisely detect and type ~80% of copy number abnormalities (CNAs) of chromosome 9p24.1, which leads to PD-1 ligand overexpression and is associated with superior outcome in cHL (65, 66).

PET/CT is considered the gold standard for staging and response assessment in cHL (67, 68). At the time of diagnosis, the total metabolic tumor volume (TMTV) represents a functional and quantitative parameter that strongly predicts the outcome in cHL (69–71). Interestingly, the amount of ctDNA in plasma correlates with TMTV, indicating that ctDNA quantification may be coupled to TMTV to better predict HL outcome at the time of diagnosis (42). In addition to baseline assessment, interim PET/CT scan after two cycles of ABVD in advanced HL represents a pivotal timepoint in treatment tailoring. Negative PET/CT patients continue the remaining ABVD cycles; conversely, positive PET/CT patients are switched to a more intensified regimen. However, a meta-analysis demonstrated a certain degree of inaccuracy of this application (72), and the combination of PET/CT scan with ctDNA analysis may fill this gap. Spina et al. observed that a >2-log reduction (DLBCL threshold) in ctDNA load after two chemotherapy courses is associated with complete response and cure in cHL. Vice versa, a drop of <2-log in ctDNA after two chemotherapy courses is associated with progression and shorter survival (32). Therefore, quantification of ctDNA complements interim PET/CT in determining residual disease in cHL. Indeed, cured patients who were inconsistently judged as interim PET/CT-positive had a >2-log drop in ctDNA, whereas relapsing patients who were inconsistently judged as interim PET/CT-negative had a less than 2-log drop in ctDNA.

### Myeloid neoplasms

The diagnosis of most myeloid neoplasms is based on morphological, immunophenotypic, and molecular characterization of bone marrow (BM) aspirates and biopsies. In addition, MRD monitoring in these neoplasms, with the exception of chronic myeloid leukemia, is currently based on sequential results obtained from BM (73). Initial studies demonstrated that cfDNA in patients with myeloid neoplasms is higher than in healthy controls, and it can be used as a reliable tool for the identification of genomic abnormalities specific to myeloid neoplasm (74, 75).

The first evidence of cfDNA value in myeloid neoplasms is derived from acute myeloid leukemia (AML) (76). More recently, targeted NGS of ctDNA has been demonstrated to detect clinically relevant mutations missed by conventional BM analysis, thus providing a complementary tool for the assessment and monitoring of AML patients (77). MRD monitoring in AML on ctDNA has also been evaluated in AML patients after allogeneic hematopoietic stem cell transplantation (alloSCT). After identifying driver mutations in 51 patients using NGS, at least one personalized digital polymerase chain reaction assay per case was designed to evaluate MRD. By analyzing multiple timepoints after alloSCT, the persistence of mutations in ctDNA over time was associated with shorter outcomes (78).

Similar approaches have also been used in myelodysplastic syndrome (MDS) patients. Serial ctDNA monitoring using digital PCR allows the detection and tracking of both driver mutations and karyotypic abnormalities during treatment and can anticipate therapy failure in MDS (79, 80). A more recent study evaluated the molecular and cytogenetic profile of MDS by NGS on ctDNA and compared the results to paired bone BM DNA samples. The mutational profile on ctDNA showed 92.1% of concordance with BM, and also the variant allele frequency of ctDNA correlated with that identified in BM. NGS on ctDNA and microarrays were highly concordant in detecting chromosomal alterations, and all cytogenetic aberrations detected by NGS in BM DNA were also detected on ctDNA. These results suggest that the analysis of ctDNA is a promising strategy for performing molecular characterization and monitoring of patients with MDS (81).

Fewer data have been reported about the use of liquid biopsy in Philadelphia-negative myeloproliferative neoplasms (MPNs). One study showed that ctDNA levels in these diseases are higher than those in healthy controls. Additionally, higher ctDNA levels have been observed in primary myelofibrosis patients than in patients with essential thrombocythemia or polycythemia vera. Similar to other hematological tumors, ctDNA reflects the mutational profile identified in genomic DNA from peripheral blood granulocytes or from BM (82).

### Multiple myeloma

MM is characterized by the uncontrolled proliferation of atypical plasma cells (83–85). Although MM is still considered a single condition, the clinical presentation, treatment response, and survival outcomes of patients are heterogeneous and rely on specific genetic lesions, including translocations involving chromosome 14q, deletion of chromosome 17p, and amplification of chromosome 1q (86).

MM is characterized by the coexistence of heterogeneous clones in the same patient that evolve during the disease course. As in other hematological malignancies, ctDNA can detect MM-specific mutations (87–89). Similar to DLBCL, the molecular analysis on BM aspirates may not reflect the different molecular profiles of the various MM subclones (90). On these grounds, the analysis of plasma-derived ctDNA can comprehensively describe the spatial mutational heterogeneity of MM (91).

MRD is also an important prognostic marker in MM and is currently investigated by multiparametric flow cytometry (MFC) or by allele-specific oligonucleotide PCR (ASO-PCR) sequencing or by NGS to detect IgH gene rearrangements in BM aspirates (92–97). Efforts are ongoing to evaluate the potential role of ctDNA as a marker of MRD and MM (9, 98).

## Future applications

### cfDNA fragmentation patterns

The study of cfDNA fragmentation patterns in liquid biopsy, also known as “fragmentomics,” and its correlation with clinical outcome has become an active area of research in recent years (99). The concept of cfDNA fragmentomics was first introduced in 2015 (100), followed by the development of several computational and experimental approaches to measure the cfDNA fragmentation patterns in plasma (101). The fragment length of cfDNA typically shows peaks of ~166 bp or multiples, which supports the evidence that apoptosis can be the main mechanism of cfDNA release. Patients with cancer present ctDNA molecules with a shorter size distribution than the background cfDNA mainly derived from hematopoietic cells (102–105). It was recently demonstrated that ctDNA fragment lengths of lymphoma patients may also vary in each individual with a correlation with disease stage. Moreover, the fragmentation patterns predict outcomes in DLBCL, therefore pointing at fragmentomics as a novel disease prognostic biomarker (44) (**Figure 1**).

### Epigenetic features

Tumor-specific DNA methylation changes are an important regulatory mechanism of gene expression that occur early in neoplastic development (106). These changes can be potentially detected in plasma even before a clinical diagnosis of cancer (107). Epigenetic alteration-based cfDNA sequencing exploits the entire pool of cfDNA rather than limiting the analysis to somatically mutated ctDNA only (108). Furthermore, epigenetic sequencing has been considered a promising alternative considering that methylation sites are scattered and ubiquitous across the human genome (109).

In the context of hematological malignancies, abnormal methylation patterns detected in cfDNA are associated with poor outcomes in DLBCL (110, 111). Recently, aberrant changes in 5-hydroxymethylcytosine (5hmC), a unique epigenetic feature in many cancers, have been identified in plasma cfDNA as an emerging and more specific biomarker for AML diagnosis and prognosis (112–114). In addition, specific DNA methylation patterns can accurately determine the tumor type from cfDNA, thus allowing a non-invasive cancer classification (112, 115–117). Conceivably, the integration of epigenetic and mutational analyses of ctDNA molecules is a promising approach to better characterize tumors (**Figure 1**).

## Clinical trials

Since ctDNA load represents a powerful prognostic biomarker for treatment tailoring, the next step essential for the introduction of ctDNA analysis in clinical practice is its evaluation in prospective clinical trials. Several ongoing clinical trials incorporate ctDNA analysis to guide treatment choices (**Table 1**) (https://clinicaltrials.gov/).

**Table 1:** Clinical trials using ctDNA for treatment tailoring.

Agent	Method(s)	Phase	Condition	Identifier
Acalabrutinib	ctDNA PET/CT	II	DLBCL	NCT04604067
Nivolumab	ctDNA	II	PCNSL	NCT04401774
R-CHOP	FDG-PET MRD	II	Untreated DLBCL	NCT03758989
Nivolumab	ctDNA	I	R/R DLBCL	NCT03311958

ctDNA, circulating tumor DNA; PET/CT, positron emission tomography/computed tomography; R-CHOP, rituximab, cyclophosphamide, doxorubicin hydrochloride (hydroxydaunorubicin), vincristine sulfate (Oncovin), and prednisone; FDG-PET, fluorodeoxyglucose positron emission tomography; MRD, minimal residual disease; PCNSL, primary central nervous system lymphoma; R/R, relapsed/refractory.

The phase II SAKK (NCT04604067) trial aims to evaluate a PET/CT and ctDNA-oriented therapy in DLBCL in order to test whether the addition of acalabrutinib to R-CHOP may improve the PFS in DLBCL patients harboring the *MYD88* L265P and/or *CD79A/B* mutations or in patients who have positive PET/CT and no molecular response (<2log_10_ reduction of ctDNA) after two courses of R-CHOP.

The phase II NCT04401774 trial is an open-label trial for patients who have completed first-line high-dose methotrexate-based chemotherapy for primary central nervous system lymphoma (PCNSL) but who have persistent ctDNA in their CSF after treatment despite radiologic response. The aim of this trial is to test whether nivolumab maintenance is safe and prevents or postpones overt clinical relapses.

The phase II NCT03758989 trial aims to measure the levels of ctDNA in patients with early-stage DLBCL to assess the change in ctDNA during treatment in order to prospectively identify biomarkers of treatment failure and to use ctDNA as a future tool for response-adapted therapy. Another objective of this study is to determine the correlation between FDG-PET and MRD, measured by ctDNA.

Patients with relapsed or refractory (R/R) DLBCL usually experience a dismal outcome, and the persistence of ctDNA after first-line chemoimmunotherapy represents a powerful biomarker of early relapse. In this context, the phase I NCT03311958 trial will explore the role of nivolumab. Patients positive for ctDNA after first-line chemoimmunotherapy will be treated with nivolumab for a period of 2 years to avoid complete relapse.

## Conclusions

The analysis of ctDNA from the liquid biopsy is being increasingly investigated in hematological malignancies as a reliable approach for tumor genotyping, outcome prediction, and disease monitoring during the course of therapy. Modern technologies have allowed the integration of standard molecular profiling with liquid biopsy for biomarker detection and analysis. The analysis of ctDNA by CAPP-seq and by the highly sensitive PhasED-seq in DLBCL and HL is a consolidated technique that allows to predict outcomes at baseline and to evaluate minimal residual disease after chemoimmunotherapy, thus anticipating clinical relapses. Different interventional clinical trials that use ctDNA analysis for treatment tailoring are ongoing. However, these high-throughput technologies often provide data not easily reproducible across laboratories, and the standardization and validation of these assays will be essential before their introduction into clinical practice.

Most efforts of liquid biopsy in hematological malignancies have been carried out by the analysis of gene mutations using the CAPP-seq and PhasED-seq methods. Liquid biopsy, however, could be also exploited to analyze other clues of the disease, including copy number abnormalities, fragmentomics, and epigenetic patterns of ctDNA that, coupled to novel statistical methods and innovative machine learning approaches, may further improve the molecular characterization of hematological diseases.

Overall, ctDNA analysis on the liquid biopsy represents a step forward toward precision medicine in patients with hematological malignancies, especially in patients with DLBCL and cHL. The combination of ctDNA dynamics with PET/CT scans at interim timepoints may further improve outcome prediction and treatment tailoring during the course of therapy. Patients with no evidence of residual disease may reduce treatment intensity; conversely, patients with persistent disease may benefit from treatment intensification. Ongoing clinical trials will answer these important questions in the near future.

## Author contributions

All authors contributed to the article and approved the submitted version.
